# Intra- and Inter-Instrument Reliability of the Actiwatch 4 Accelerometer in a Mechanical Laboratory Setting

**DOI:** 10.2478/v10078-012-0002-z

**Published:** 2012-04-03

**Authors:** Ashley C. Routen, Dominic Upton, Martin G. Edwards, Derek M. Peters

**Affiliations:** 1 Institute of Sport and Exercise Science, University of Worcester, Worcester, Worcestershire, UK.; 2 Institute of Health and Society, University of Worcester, Worcester, Worcestershire, UK.; 3 Institut des Sciences Psychologiques, Université catholique de Louvain, Place du Cardinal Mercier, Louvain-la-Neuve, Belgium.; 4 Faculty of Health and Sport Sciences, University of Agder, Kristiansand, Norway.

**Keywords:** physical activity, activity monitor, measurement, repeatability, accelerometer

## Abstract

This study aimed to quantify the intra-and inter-instrument reliability of the Actiwatch 4 accelerometer (AW4) in a mechanical setting. Twenty seven AW4 were attached to an isokinetic dynamometer and subjected to angular acceleration for 30 min at 50 deg/sec representing moderate intensity (MPA condition) and 200 deg/sec representing vigorous intensity (VPA condition), with a repeat trial conducted. Reliability was assessed using coefficient of variation (CV), absolute percent error (APE), and intraclass correlation coefficients (ICC). Mean AW4 activity counts displayed acceptable reliability according to CV in both conditions (ConMPA: CVintra = 4.6%, APEintra = 6.6%, CVinter = 6.4%, APEinter = 5.2%; ConVPA: CVintra = 3.9%, APEintra = 5.6%, CVinter = 5.9%, APEinter = 4.7%). When counts were re-categorised into minutes of MPA and VPA, lower CV values were observed (ConMPA: CVintra = 3.2%, APEintra = 4.5%, CVinter = 4.3%, APEinter = 3.2%; ConVPA: CVintra = 0.0%, APEintra = 0.0%, CVinter = 0.0%, APEinter = 0.0%). When activity counts were re-categorised as minutes of MVPA, excellent reliability was observed (CVintra, APEintra, CVinter, and APEinter = 0.0%) in both conditions. Mean AW4 activity counts exhibit reliability statistics comparable to other accelerometers. Reliability is improved when activity counts are re-categorised as time spent in MPA and VPA, with greatest reliability obtained when counts are recategorised as time spent in MVPA. As MVPA is the subcomponent of physical activity most associated with health benefits it would appear that the AW4 is reliable for measuring time spent in this health enhancing intensity category, at least from testing in a mechanical laboratory setting.

## Introduction

Accelerometry has become an increasingly popular method to objectively measure physical activity (Rowlands, 2007; Skalik et al., 2009). Evidence from studies employing accelerometers have been used to better identify relationships between physical activity and health outcomes (Andersen et al., 2006; Ness et al., 2007). This is in part due to the increased measurement accuracy and precision afforded through the use of accelerometers compared to subjective measures (Corder et al., 2008). Despite the widespread use of accelerometers and the increase in monitor technology, information on many aspects of these devices is still limited (Esliger and Tremblay, 2006). The majority of research using accelerometers has focused upon the development of energy expenditure prediction equations and intensity cut-off values. In contrast to the great number of accelerometer validity investigations, and despite calls from review papers (Ward et al., 2005; Welk, 2005), research on the reliability of some accelerometer models remains limited.

Studies that have previously investigated the reliability of accelerometer devices can be divided into participant mounted (either laboratory based or free-living protocols) or laboratory based mechanical reliability studies (Fairweather et al., 1999; Metcalf et al., 2002; Brage et al., 2003; Powell et al., 2003; Powell and Rowlands, 2004; Esliger and Tremblay, 2006; McClain et al., 2007; Krasnoff et al., 2008). The investigations focusing upon mechanical laboratory experiments have used various apparatus to accelerate the accelerometer devices. These include rotating wheels (Brage et al., 2003), turntables (Metcalf et al., 2002), and hydraulic shaker plates (Powell et al., 2003; Esliger and Tremblay, 2006). In comparison to human experiments mechanical devices have several advantages, such as the large number of accelerations that can be generated, the ability to record data from multiple instruments simultaneously, and the high reproducibility of oscillations between trials (Esliger and Tremblay, 2006).

Accelerometer reliability studies have to date focused solely upon reproducibility of raw activity counts. However, most published research investigating the relationship between accelerometer measured physical activity and health outcomes is presented using derived activity variables, such as time spent above a given intensity level e.g. minutes spent in MVPA. Only a single study to date has investigated the ability of an accelerometer to reliably classify raw activity counts into a derived intensity variable. McClain et al. (2007) examined the inter-instrument reliability of concurrently worn (left hip and right hip sites) Actigraph accelerometers (7164) during free living conditions. They assessed both raw and derived variables and found that inter-instrument reliability of the Actigraph for classifying time spent in MVPA was acceptable (CV = 3.7%, APE = 4.9%, and ICC *r* = 0.99). McClain et al. (2007) concluded that MVPA may be the best derived physical activity intensity variable to use due to the reduced likelihood of count misclassification between the moderate and vigorous categories as a consequence of using a composite variable; that is moderate + vigorous activity.

The Actiwatch (AW) accelerometer (one of the few wrist-worn accelerometers currently available) has been validated against energy expenditure in children, with energy expenditure prediction equations and intensity-cutpoints also being derived (Puyau et al., 2002; 2004). Despite the AW’s validation as an activity monitor there have been no published examinations of either the intra or inter-instrument reliability, and therefore the reproducibility of this accelerometer-based physical activity monitor is unknown. The purpose of this study was to quantify the intraand inter-instrument reliability of the Actiwatch 4 when accelerated under conditions representative of moderate and vigorous intensity in a mechanical laboratory setting.

## Material and Methods

### Instrumentation

#### Actiwatch 4 Accelerometer (AW4)

The AW4 is a small (37 × 29 × 10 mm) wrist worn accelerometer which weighs 16 g and has a random access memory (RAM) capacity of 64 kb. It constitutes of a rectangular piezoelectric bimorph plate and seismic mass. It is omnidirectional, but is most sensitive in the vertical axis. This technology detects the peak amplitude of movement acceleration and generates a transient voltage signal proportional to the rate of acceleration (Cambridge Neurotechnology, 2007). The raw digital voltage strings are converted to activity counts, with the peak count being selected for each individual second. Peak activity counts are integrated (and recorded) during a user-specified time interval (epoch), which ranges from 2 seconds to 15 minutes. The device has a sampling frequency of 32 Hz and collects motion in the frequency range of 0.5–7.0 Hz (Chen and Bassett, 2005; Cambridge Neurotechnology, 2007).

### CSMi Isokinetic Dynamometer

All testing was completed using a CSMi Isokinetic Dynamometer (Computer Sports Medicine Inc., Stoughton, MA, US). The dynamometer was selected as it can produce constant motion at speeds ranging from 1 to 500 deg x sec^−1^, with a total range of motion of 360°.

### Experimental Procedure

#### Pilot Tests

To select the test speeds that were representative of moderate and vigorous intensity, five Actiwatch 4 units were attached to the knee/hip arm adapter of an Isokinetic dynamometer and were accelerated at six different test speeds (50, 100, 150, 200, 250, 350 deg × sec^−1^). The units were set to record at 10 second epochs, as this is the maximum resolution for 7 day data capture in the AW4. The mean of the five units was compared to published one-minute intensity cut-points (Puyau et al., 2004), which were divided by six to provide a moderate intensity threshold of 117–416 cts × 10s^−1^, and a vigorous intensity threshold of ≥417 cts × 10s^−1^ for the 10 second epoch data captured. The test speed of 50 deg × sec^−1^ (0.55 Hz) produced ∼300 cts × 10s^−1^ and was therefore selected as the MPA representative ConMPA. The test speed of 200 deg × sec^−1^ (2.2 Hz) produced ∼600 cts × 10s^−1^ and was therefore selected as the VPA representative ConVPA. Twenty seven Actiwatch accelerometers were intialised to collect data using 10 second epochs. Up to five accelerometer units at a time were mounted to the knee/hip adapter of the isokinetic dynamometer. They were positioned perpendicular to the floor, maximising time spent in the vertical axis. The dynamometer was set to move through a 90° range of motion, and each unit was accelerated for 30 minutes at 50 deg × sec^−1^ (ConMPA), and 30 minutes at 200deg × sec1 (ConVPA). An identical repeat trial was conducted in each condition (Trial 1, Trial 2). All study procedures were approved by the Ethical Advisory Committee of the Institute of Sport and Exercise Science, University of Worcester, UK.

### Data treatment and statistical analysis

Data were first imported into Microsoft Excel and the recorded condition start and end times were identified. The first and last minute of each unit’s data was deleted, to ensure that no spurious results were included in the dataset, leaving raw data for 28 minutes per condition (Esliger and Tremblay, 2006). The data were imported into SPSS for Windows Version 17.0 (SPSS Inc., Chicago, IL) for further analysis. Mean activity counts (cts × 10s^−1^), and derived variables of time spent in MPA for ConMPA, time in VPA for ConVPA, and time spent in MVPA in both conditions were calculated from the raw data in each accelerometer for Condition MPA, Trial 1: (ConMPA_Tr1), Condition MPA, Trial 2 (ConMPA_Tr2), Condition VPA, Trial 1 (ConVPA_Tr1), and Condition VPA, Trial 2 (ConVPA_Tr2).

### Intra-instrument reliability

To explore the reliability within accelerometers five methods were used: (a) the standard deviation (SD) between trials; (b) the coefficient of variation (CV_intra_) for each condition between trials calculated by dividing the SD of the individual unit mean (between trials 1 and 2), by the individual unit mean (trial 1 mean + trial 2 mean/2), multiplied by one hundred [SD/Mean × 100] (c) the APE_intra_, calculated by subtracting the individual unit mean for trial 2 (trial 2 mean - trial 1 mean) from trial 1, the product of which was divided by the overall trial mean (trial 1 mean + trial 2 mean/2), multiplied by one hundred [(Trial 2-Trial 1)/Overall Trial × 100]; (d) by paired samples t-tests on the differences in unit means, between trials to determine systematic bias; and (e) with the Intraclass correlation coefficient (ICC) of absolute agreement. The alpha level was set at *p*<0.05 for all tests. If a difference was found Cohen’s *d* was calculated (small = 0.2, medium = 0.5, large = 0.8, Cohen, 1988) as an estimate of effect size.

### Inter-instrument reliability

Reliability between accelerometers was examined as per above, excluding t-tests and ICC. CV_inter_ was calculated by dividing the SD between individual unit means (trial 1 mean + trial 2 mean/2), by the overall group mean (trial 1 group mean + trial 2 group mean/2), multiplied by one hundred [SD/Mean × 100]. APE_inter_ was calculated by subtracting the individual unit mean (trial 1 mean + trial 2 mean/2) from the overall group mean (trial 1 group mean + trial 2 group mean/2), the product of which was divided by the overall group mean, multiplied by one hundred [(Individual-Group)/Group × 100].

## Results

Descriptive data for mean activity counts and reliability statistics for both conditions and trials are presented in Table 1. Descriptive data for mean time spent in physical activity intensity categories and reliability statistics for both conditions and trials are presented in Table 2.

From Table 1 the CV_intra_ for mean activity counts was 4.6% for the MPA condition and 3.9% for the VPA condition, the combined mean of both conditions being CV_intra_ = 4.3%. Mean activity counts per epoch were greater in ConMPA_Tr1 compared to ConMPA_Tr2 (Mean ± SD: 329 ± 24 vs. 310 ± 21 cts × 10s^−1^, *t*(26) = 5.2, *p* = 0.01, *d* = 0.8), and were greater in ConVPA_Tr1 compared to ConVPA_Tr2 (Mean ± SD: 621 ± 44 vs. 602 ± 38 cts × 10s^−1^, t(26) = 2.3, *p* = 0.03, *d* = 0.5). The APE_intra_ was 6.6% for the MPA condition and 5.6% for the VPA condition, the combined mean of both conditions being APE_intra_ = 6.1%.

From Table 2, the CV_intra_ for minutes of MPA was 3.2% for the MPA condition, and for minutes of VPA in the VPA condition was 0.0%. The CV_intra_ for minutes of MVPA in both conditions was 0.0%. Time spent in MPA was greater in ConMPA_Tr2 compared to ConMPA_Tr1 (27.6 ± 0.7 vs. 26.5 ± 1.8 min, *t*(26) = −4.0, *p* = 0.01, *d* = 0.7). There was no difference in time spent in VPA between ConVPA_Tr1 and ConVPA_Tr2 (28.0 ± 0.0 vs. 28.0 ± 0.0 mins). No differences were found in time spent in MVPA between ConMPA_Tr1 and ConMPA_Tr2 or between ConVPA_Tr1 and ConVPA_Tr2.

The APE_intra_ was 4.5% for the MPA condition and 0.0% for the VPA condition, the combined mean of both conditions being APE_intra_ = 2.2%.The ICC for mean activity counts between ConMPA_Tr1 and ConMPA_Tr2 was 0.67 (*F*(26)=4.9, *p* = 0.01). The ICC for mean activity counts between ConVPA_Tr1 and ConVPA_Tr2 was 0.64 (*F*(26) = 3.0, *p*= 0.01). The ICC for minutes of MPA between ConMPA_Tr1 and ConMPA_Tr2 was 0.51 (*F*(26)= 2.6, *p* = 0.01).

From Table 1 the CV_inter_ for mean activity counts was 6.4% for the MPA condition and 5.9% for the VPA condition, the combined mean of both conditions being CV_inter_ = 6.1%. The APE_inter_ for mean activity counts was 5.2% for the MPA condition, and 4.7% for the VPA condition, the overall mean of both conditions being 5.0%. From Table 2, the CV_inter_ for minutes of MPA was 4.3% in the MPA condition, and for time spent in VPA in the VPA condition was 0.0%. The CV_inter_ for MVPA in both conditions was 0.0%. APE for time spent in MPA was 3.2% for the MPA condition and for time spent in VPA in the VPA condition was 0.0%. The APE_inter_ for MVPA in both conditions was 0.0%.

## Discussion

This is the first study to evaluate the reliability of the Actiwatch 4 and only the second study to examine the reliability of derived activity variables in an accelerometer. These data demonstrate the Actiwatch 4 to have acceptable intra-instrument reliability for raw mean activity counts according to the CV values. In ConMPA the CV_intra_ was 4.6%, which is of similar magnitude to the 3.2% observed in the Actigraph 7164 (Esliger and Tremblay, 2006), the 1.4% observed in the CSA (at present known as Actigraph 7164) (Metcalf et al., 2002), and the 1.8% observed in the RT3 (Krasnoff et al., 2008). The higher CV_intra_ observed in the present study may be due to differences in the experimental protocol (i.e. test duration, tests speeds, number of units tested, mechanical acceleration equipment) and the use of other accelerometer models that differ from the AW4 in device sensitivity, axes of measurement, frequency range and signal weighting.

A significant difference however was found for activity counts between trials, with systematically lower activity counts produced in trial 2 in both conditions. This may reflect both systematic bias and random trial related error such as AW4 battery discharge, and resonance in the experimental setup between trials. The significant difference between trials, was however only ∼20 counts between trials over a 28 minute test condition, which on average reflects approximately 5% of the combined mean of all trials.

In the VPA condition the intra-instrument variance was reduced (CV_intra_ = 3.9%), the implication being that raw counts show some variance within units, becoming less variable as the test speed increased. The intra-instrument reliability of raw activity data was greater than inter-instrument reliability, which is consistent with the findings of prior studies (Powell et al., 2003; Esliger and Tremblay, 2006; Krasnoff et al., 2008). The CV_inter_ was observed as 6.4% in the MPA condition and 5.9% in the VPA condition, again higher in the MPA condition. Krasnoff et al. (2008) found fairly high CV_inter_ (9.5–34.7%) among RT3 accelerometer units oscillated on a hydraulic shaker table, believed to be attributed to the devices wide frequency range (Esliger and Tremblay, 2006). Similarly Esliger and Tremblay (2006) observed a mean CV_inter_ of 8.6% between Actigraph units that were accelerated at varying speeds, therefore the findings of the present study in the Actiwatch 4 are aligned with previously published parameters of inter-instrument variability in other accelerometer models.

The ICC between trials for both conditions ranged from 0.51–0.67, which is lower than the values (0.84–0.93) reported by Metcalf et al. (2002) and (0.91–0.98) by Esliger and Tremblay (2006). However, the units used in this study were re-conditioned from previous clinical trials and displayed heterogeneity in between trial variance, potentially reducing the overall ICC. The importance of the ICC per se is however limited as it gives no indication of the magnitude of disagreement between trials (Metcalf et al., 2002), which as discussed above was found to be practically speaking ‘insignificant’.

Intra-and inter-instrument variability in raw and derived variables were greater in the moderate intensity condition compared to the vigorous intensity condition. This is congruent with data from previous studies showing an inverse relationship between test speeds (intensity of work) and variability in raw activity counts produced by the Actical (Esliger and Tremblay, 2006), and between frequency/acceleration and variability in raw activity counts produced by the RT3 (Powell et al, 2003). These data show that the magnitude of error in the Actiwatch differed between test speeds, such that measurement error in the Actiwatch may depend upon the magnitude of the acceleration measured.

As a calibration check Esliger and Tremblay (2006) suggest an example *a priori* calibration variability limit of an APE_inter_ of ≤ 5% may be set, for the selection of reliable units. If this had been applied to the units used in the current study, 14 of the 27 (52%) units would have been rejected as unreliable from the outset. Individually, these 14 units displayed bias (both under and overestimation) in mean activity counts, when compared to the mean value of the entire sample. However by assessing inter-instrument reliability between the separate derived variables of time spent in physical activity intensity categories as opposed to using mean activity counts, the discrepancy between the individual units and the sample mean expressed as APE was reduced (Mean APE Raw vs. Derived: Con MPA: 5.2% vs. 1.6%, Con VPA: 4.7% vs. 0.0%) to under the suggested 5% inclusion threshold. Further, when examining the intra-instrument reliability, CV_intra_ reduced from 4.6% for raw variables to 1.6% for derived variables in Condition MPA, and from 3.9% to 0% in Condition VPA. Therefore when applying the 5% reliability threshold to the separate intensity derived variables in both conditions all 27 units were deemed acceptable for research use.

As noted prior there was a significant difference in activity count output between trials in Condition MPA, resulting in a difference of 1.1 (decimal) minutes of MPA between trials. Whilst significant in this mechanical laboratory setting, in vivo the clinical significance of this systematic bias may be small. Further, 1.5 (decimal) minutes of MPA were misclassified as VPA in ConMPA_Tr1, with 0.4 minutes misclassified as VPA in ConMPA_Tr2. Clearly therefore the use of separate intensity categories may result in the misclassification of activity counts, as counts are placed into discrete categories i.e. moderate or vigorous activity (McClain et al., 2007). When combining MPA and VPA into the more practically significant MVPA, intra-and-inter instrument reliability was improved in both conditions (CV = 0.0%, APE = 0.0%) in agreement with the findings of McClain et al. (2007).

On this basis a pragmatic applied research decision should be made. Whilst it is clear that there are discrepancies between mean activity counts, both intra- and -inter- unit reliability is clearly improved by using derived variables. Those units that may have been excluded on the basis of an APE_inter_ >5% in mean activity counts (Esliger and Tremblay, 2006), were deemed acceptable for the purposes of research when running a calibration check using derived activity data. As the majority of researchers and practitioners use derived variables to give biological meaning to an otherwise arbitrary accelerometer output (Corder et al., 2008) and as the use of MVPA as an outcome measure has become increasingly common (McClain et al., 2007), representing the minimum intensity of physical activity that both adults and children are recommended to accrue according to current physical activity guidelines (O’Donavon et al., 2010), the current study would suggest that when using the Actiwatch 4, raw activity counts should be categorised into minutes of MVPA to improve data reliability.

The observed variation in AW4 output between units suggests that when employing these devices longitudinally participants should wear the identical device to ensure that artificial differences between time-points do not manifest. It is important that researchers test the precision of all wearable motion sensors prior to use. This should be conducted using a mechanical device which can replicate test speeds that are physiologically relevant, ensuring that identification of (in)variance can solely be attributed to the monitor, and not to within-subject biological variation (i.e. gait biomechanics and monitor positioning associated with body composition and clothing).

## Conclusions

In summary, in a mechanical laboratory setting both intra-instrument and inter-instrument reliability of raw activity counts was acceptable, with greatest variance observed in the moderate representative condition. When derived variables of time spent in MPA, VPA and MVPA were used, greater reliability was observed in both conditions. It is apparent that the AW4 can reliably categorise raw activity counts into the health enhancing intensity category of MVPA when accelerated at speeds producing a count output of at least moderate intensity in a mechanical laboratory setting. Therefore, dependent upon the research question (and if separate intensity categories are not of interest) future research using the AW4 should report the combined category of time spent in MVPA as opposed to separate categories of moderate and vigorous activity to increase data reliability.

## Figures and Tables

**Table 1:** Mean values of raw Actiwatch count output, and intraand inter-instrument reliability statistics

**Conditions**			**Intra-Instrument Reliability**			**Inter-Instrument Reliability**		

**Trial**	**Condition**	**Counts**	**SD**	**CV**	**APE**	**SD**	**CV**	**APE**
**1**	**MPA**	329^*^	14.8	4.6	6.6	20.4	6.4	5.2
**2**	**MPA**	310^*^						
**1**	**VPA**	621^†^	24.3	3.9	5.6	35.9	5.9	4.7
**2**	**VPA**	602^†^						
**Overall Mean**		**465**	**19.6**	**4.3**	**6.1**	**28.2**	**6.1**	**5.0**

* Difference between trials (p = 0.01).

† Difference between trials (p = 0.03).

SD = standard deviation, CV = coefficient of variation, APE = absolute percent error

**Table 2:** Mean values of derived physical activity category variables, and intra- and inter-instrument reliability statistics

**Conditions**				**Intra-Instrument Reliability**			**Inter-Instrument Reliability**		

**Trial**	**Condition**	**Intensity Category**	**Minutes**	**SD**	**CV**	**APE**	**SD**	**CV**	**APE**
**1**	**MPA**	MOD	26.5^*^	0.8	3.2	4.5	1.2	4.3	3.2
**2**	**MPA**	MOD	27.6^*^						
**1**	**MPA**	MVPA	28.0	0.0	0.0	0.0	0.0	0.0	0.0
**2**	**MPA**	MVPA	28.0						
**1**	**VPA**	VIG	28.0	0.0	0.0	0.0	0.0	0.0	0.0
**2**	**VPA**	VIG	28.0						
**1**	**VPA**	MVPA	28.0	0.0	0.0	0.0	0.0	0.0	0.0
**2**	**VPA**	MVPA	28.0						

* Difference between trials (p = 0.01). SD = standard deviation, CV = coefficient of variation, APE = absolute percent error, MOD = moderate intensity, MVPA = moderate to vigorous intensity, VIG = vigorous intensity
